# Efficacy and reinfection with soil-transmitted helminths 18-weeks post-treatment with albendazole-ivermectin, albendazole-mebendazole, albendazole-oxantel pamoate and mebendazole

**DOI:** 10.1186/s13071-016-1406-8

**Published:** 2016-03-02

**Authors:** Benjamin Speich, Wendelin Moser, Said M. Ali, Shaali M. Ame, Marco Albonico, Jan Hattendorf, Jennifer Keiser

**Affiliations:** Department of Medical Parasitology and Infection Biology, Swiss Tropical and Public Health Institute, Basel, Switzerland; University of Basel, Basel, Switzerland; Laboratory Division, Public Health Laboratory-Ivo de Carneri, Chake Chake, Tanzania; Ivo de Carneri Foundation, Milan, Italy; Department of Epidemiology and Public Health, Swiss Tropical and Public Health Institute, Basel, Switzerland

**Keywords:** *Trichuris trichiura*, *Ascaris lumbricoides*, Hookworm, Randomised controlled trial, Reinfection, Oxantel pamoate

## Abstract

**Background:**

Preventive chemotherapy with albendazole or mebendazole is the current strategy to control soil-transmitted helminth (STH) infections (i.e. *Ascaris lumbricoides*, hookworm and *Trichuris trichiura*). STH reinfections, in particular *A. lumbricoides* and *T. trichiura* occur rapidly after treatment with the standard drugs. However, their low efficacy against *T. trichiura*, made an accurate assessment of reinfection patterns impossible.

**Methods:**

In 2013 a randomised controlled trial was conducted on Pemba Island, Tanzania. School-aged children diagnosed positive for *T. trichiura*, were randomly allocated to (i) albendazole-ivermectin; (ii) albendazole-mebendazole; (iii) albendazole-oxantel pamoate; or (iv) mebendazole. Here we report the efficacy [cure rates (CR) and egg-reduction rates (ERR)], reinfection rates and new infections determined 18 weeks post-treatment.

**Results:**

For a total of 405 children complete baseline and follow-up data were available. Similar to the efficacy determined after 3 weeks, 18 weeks after treatment albendazole-oxantel pamoate showed a significantly higher efficacy against *T. trichiura* (CR: 54.0 %, 95 % CI: 43.7–64.0; ERR: 98.6 %, 95 % CI: 97.8–99.2) compared to the other treatment arms. Children treated with albendazole-oxantel pamoate or albendazole-ivermectin had fewer moderate infections compared to children treated with albendazole. The reinfection rates 18 weeks post-treatment among all treatment arms were 37.2 % for *T. trichiura* (95 % CI: 28.3–46.8), 34.6 % for *A. lumbricoides* (95 % CI: 27.3–42.3) and 25.0 % for hookworms (95 % CI: 15.5–36.6).

**Conclusion:**

The moderate reinfection rates with STHs 18 weeks post-treatment support the concept of regular anthelminthic treatment in highly endemic settings. Combination chemotherapy might achieve decreased morbidity in children since in the albendazole plus oxantel pamoate and albendazole plus ivermectin treatment arms only few moderate *T. trichiura* infections remained. Further trials should investigate the long term efficacy of albendazole-oxantel pamoate (i.e. 6 and 12 month post-treatment) and after several rounds of treatment in order to develop recommendations for appropriate control approaches for STH infections.

**Trial registration:**

Current Controlled Trials ISRCTN80245406

**Electronic supplementary material:**

The online version of this article (doi:10.1186/s13071-016-1406-8) contains supplementary material, which is available to authorized users.

## Background

The most common soil-transmitted helminths (STH; *Ascaris lumbricoides*, hookworms and *Trichuris trichiura*) infect approximately 1.5 billion people [1] worldwide with the highest prevalences in Asia and Africa. School-aged children living in the least developed settings, lacking clean water and sanitation facilities are primarily affected by *A. lumbricoides* and *T. trichiura*, while hookworm infections mostly occur in adults [2, 3]. Untreated, chronically infected children might suffer from anemia, malnutrition and impairments in cognitive and physical development [2]. The burden of soil-transmitted helminthiasis has been estimated as 5.3 million disability-adjusted life years [1]. Large scale distribution of anthelminthic drugs without prior diagnosis (“preventive chemotherapy”) mainly given to school-aged children, is the current strategy to control morbidity [4]. The most common anthelminthic drugs are albendazole and mebendazole [5]. Both drugs have high efficacy against *A. lumbricoides*, while only albendazole reveals a good performance in the treatment of hookworm infections. For the treatment of *T. trichiura* both drugs show poor cure rates in single-dose regimen [6].

Preventive chemotherapy does not avert reinfections as demonstrated in earlier studies [7, 8]. Six to 12 months after treatment with albendazole or mebendazole, the prevalence of *A. lumbricoides* reached the pretreatment level [9–11], while hookworm reinfection is slow [9]. However, it is difficult to accurately estimate the reinfection rate of *T. trichiura* since, as mentioned above, the efficacy of the benzimidazoles against *T. trichiura* is low [6], in particular when children suffer from high infection intensity [12].

In a clinical trial conducted in 2013 on Pemba Island, Tanzania, we examined the efficacy of three drug combinations (i.e. albendazole-ivermectin, albendazole-mebendazole, and albendazole-oxantel pamoate) versus mebendazole against *T. trichiura* and concomitant STH infections [13]. In brief, the combination albendazole-oxantel pamoate revealed a significantly higher cure rate (CR) (68.0 %) and egg-reduction rate (ERR 99.2 %) against *T. trichiura* than the other treatment regimens. This high efficacy might allow drawing better conclusions on reinfection with *T. trichiura* and ultimately to develop recommendations for control efforts.

The aim of the present study was to investigate whether the efficacy of albendazole-oxantel pamoate remains superior to the other combinations 18 weeks post-treatment and to monitor reinfection patterns of *T. trichuris*, *A. lumbricoides* and hookworms.

## Methods

### Study oversight

The presented data derive from a randomised controlled trial conducted among school-aged children on Pemba Island, Tanzania [13]. Ethical clearance was obtained from the Ministry of Health and Social Welfare in Zanzibar, Tanzania (ZAMREC, reference no. 0001/June/13) and from the ethics committee of Basel, Switzerland (EKBB reference no. 123/13). The trial was registered with www.isrctn.com (identifier: ISRCTN80245406). Prior to the study start, written informed consent was obtained from the parents or legal guardians and verbal assent from the participating children.

### Study procedures and patients

The clinical trial was conducted from September 2013 to January 2014. Study setting and trial procedures are presented elsewhere [13]. In brief, school-aged children diagnosed positive for *T. trichiura* were randomly assigned to one of the following treatment arms: (i) albendazole (400 mg) plus ivermectin (200 μg/kg); (ii) albendazole (400 mg) plus mebendazole (500 mg); (iii) albendazole (400 mg) plus oxantel pamoate (20 mg/kg); and (iv) mebendazole (500 mg). All children were invited 3 and 18 weeks after treatment to provide stool samples on two consecutive days for the diagnosis of STH infections. Duplicate Kato-Katz thick smears were prepared from each stool sample using 41.7 mg templates [14] and quantitatively examined by experienced laboratory technicians for eggs of *T. trichiura*, hookworms and *A. lumbricoides*. Slides were read under a light microscope within 60 min after preparation to avoid over-clearing of hookworm eggs [15]. Ten percent of the Kato-Katz thick smears were randomly chosen and re-examined to assure high quality of the microscopic diagnosis [16]. In case of discrepancies, the slides were read again and discussed until consensus was reached. At the end of the study, all children remaining infected from the two schools (Mchangamdogo and Shungi) received treatment according to national guidelines [17].

### Statistical analysis

Prevalence of infection with *T. trichiura*, *A. lumbricoides* and hookworms was calculated for each treatment arm at baseline, 3 and 18 weeks post-treatment for all children with a complete dataset (2 stool samples at each time point). Differences among treatment arms in CRs 3 weeks and extended CRs (extended CR: children positive at baseline and negative at both follow-ups) 18 weeks post-treatment against *T. trichiura*, *A. lumbricoides* and hookworms were assessed using logistic regression.

Geometric means (GM) for eggs per gram (EPG) were calculated by adding 1 to each count (to take the logarithm in case of EPG = 0), taking the GM and subtracting 1 (GM  =  exp ((∑ log (EPG + 1))/n) − 1) [18]. Bootstrap resampling method with 10,000 replicates was used for calculating the 95 % confidence intervals (CI) for the ERRs. We assumed non-overlapping CI as statistical significant difference in ERRs. All arithmetic means (AM) are presented in the Additional file 1: Table S1.

Reinfection rates were defined as children positive at baseline, negative 3 weeks and positive 18 weeks post-treatment. New infections were defined as children negative at baseline and 3 weeks after treatment and positive 18 weeks post-treatment. As all children were by design positive for *T. trichiura*, new infections according to our definition, were only observed for *A. lumbricoides* and hookworms. Children negative at baseline and positive 3 weeks later were not included in these calculations (excluded for *A. lumbricoides*: 4 out of 405 children; and for hookworm: 27 out of 405 children).

## Results

### Efficacy of drug combinations against *T. trichiura* 3 and 18 weeks post-treatment

**Fig. 1:**
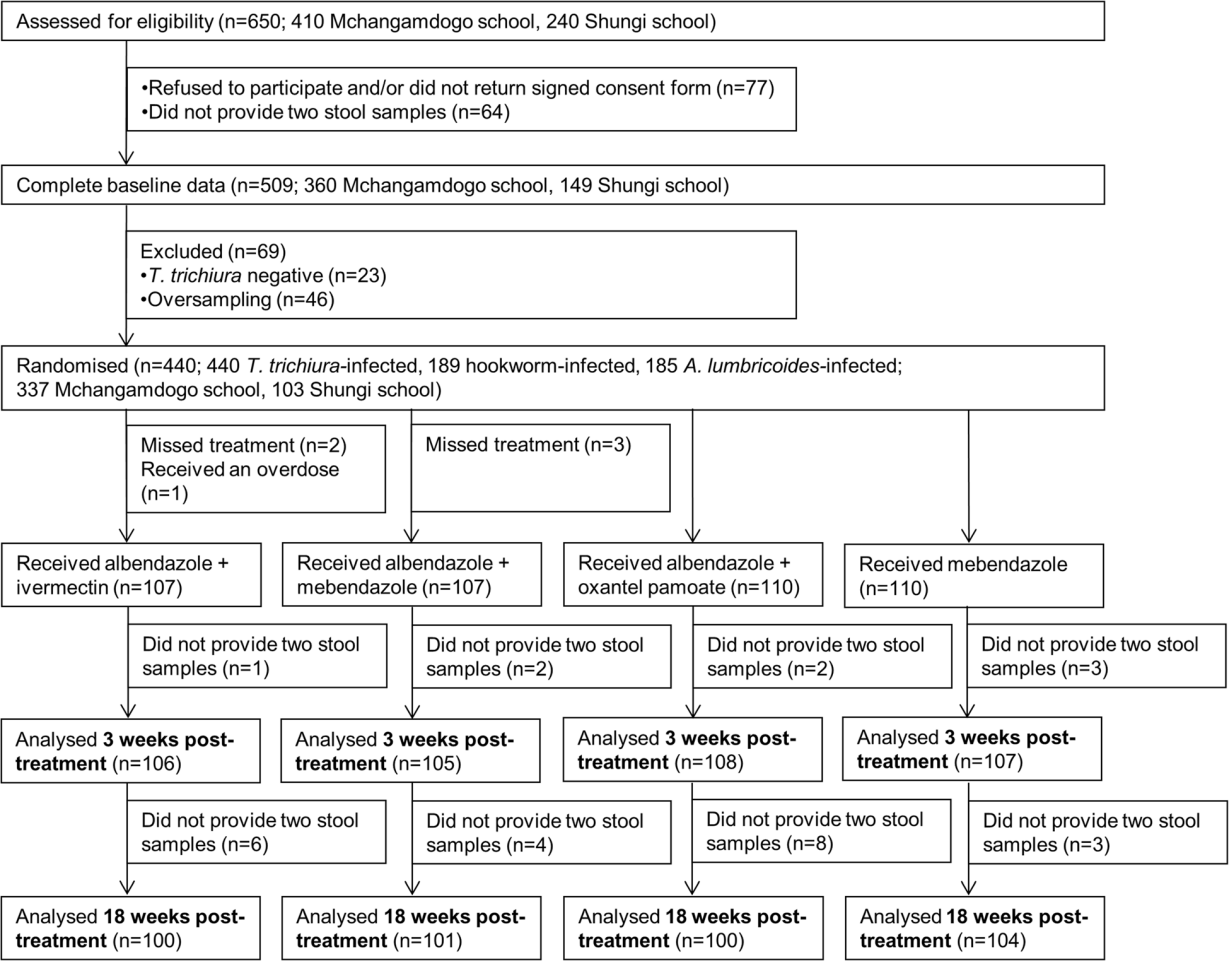
Study design flow chart. Study enrolment, randomisation and two follow-ups (3 and 18 weeks post-treatment) for the four-arm, randomised controlled trial

In total 405 children infected with *T. trichiuria* were allocated to four different treatment regimens and provided all six stool samples (i.e. duplicate stool samples at baseline, 3 and 18 weeks follow-up; Fig. 1). The efficacies 3 weeks post-treatment observed with the different treatment regimens are presented elsewhere [13] and summarised in Table 1. The CRs documented 18 weeks post-treatment were 54.0 % (43.7–64.0) for albendazole-oxantel pamoate, 20.0 % (12.7–29.2) for albendazole-ivermectin, 13.9 % (7.8–22.2) for albendazole-mebendazole and 10.6 % for mebendazole (5.4–18.1). At the second follow-up, the efficacy of the other treatment arms remained significantly lower compared to albendazole-oxantel pamoate in terms of CR (*P*-values in Table 1) and ERR (98.6, CI: 97.8–99.2) compared to the other treatments at the second follow up. Considering the arithmetic ERRs, albendazole combined with ivermectin and albendazole-oxantel pamoate were significantly higher compared to the other treatments 18 weeks post-treatment (Additional file 1: Table S1).

**Table 1:** Cure rates (CR), extended CRs, egg-reduction rates (ERR), extended ERRs and reinfection data for the four different treatments against *T. trichiura* infections. Results of baseline and 3 weeks follow-up have been reported elsewhere [13]

*Trichuris trichiura*					
	Weeks post-treatment	Albendazole – ivermectin (*n* = 100)	Albendazole – mebendazole (*n* = 101)	Albendazole – oxantel pamoate (*n* = 100)	Mebendazole (*n* = 104)
Children positive before treatment (%)		100 (100)	101 (100)	100 (100)	104 (100)
No. of children cured (CR, 95 % CI)	3 weeks	28 (28.0, 19.5–37.9, *p* < 0.001)*	9 (8.9, 4.2–16.2, *p* < 0.001)*	68 (68.0, 57.9–77.0)	8 (7.7, 3.4–14.6, *p* < 0.001)*
No. of children negative (extended CR, 95 % CI)	18 weeks	20 (20.0, 12.7–29.2, *p* < 0.001)*	14 (13.9, 7.8–22.2, *p* < 0.001)*	54 (54.0, 43.7–64.0)	11 (10.6, 5.4–18.1, *p* < 0.001)*
Geometric mean: EPG	Baseline	489.9	390.0	471.3	467.8
	3 weeks	25.0	176.0	3.7	207.8
	18 weeks	47.8	128.1	6.6	158.8
ERR (95 % CI)	3 weeks	94.9 (92.3–96.7)	54.9 (38.3–67.7)	99.2 (98.6–99.6)^a^	55.6 (40.0–68.0)
Extended ERR (95 % CI)	18 weeks	90.2 (85.3–93.6)	67.2 (51.9–77.9)	98.6 (97.8–99.2)^a^	66.1 (52.0–76.6)
No. of children positive (prevalence, 95 % CI)	Baseline	100 (100, −)	101 (100, −)	100 (100, −)	104 (100, −)
	3 weeks	72 (72.0, 62.1–80.5)	92 (91.1, 83.8–95.8)	32 (32.0, 23.0–42.1)	96 (92.3, 85.4–96.6)
	18 weeks	80 (80.0, 70.8–87.3)	87 (86.1, 77.8–92.2)	46 (46.0, 36.0–56.3)	93 (89.4, 81.9–94.6)
Reinfections (%, 95 % CI)	18 weeks	15/28 (53.6, 33.9–72.5)	2/9 (22.2, 2.8–60.0)	21/68 (30.9, 20.2–43.6)	4/8 (50.0, 15.7–84.3)

Data are n (%, 95 % CI) unless otherwise indicated. EPG = egg per gram of stool. *Significantly lower CR compared to albendazole-oxantel pamoate (*P*-values derived from logistic regression)

^a^Significantly higher ERR compared to other treatment arms (no overlapping confidence interval assumption)

**Fig. 2:**
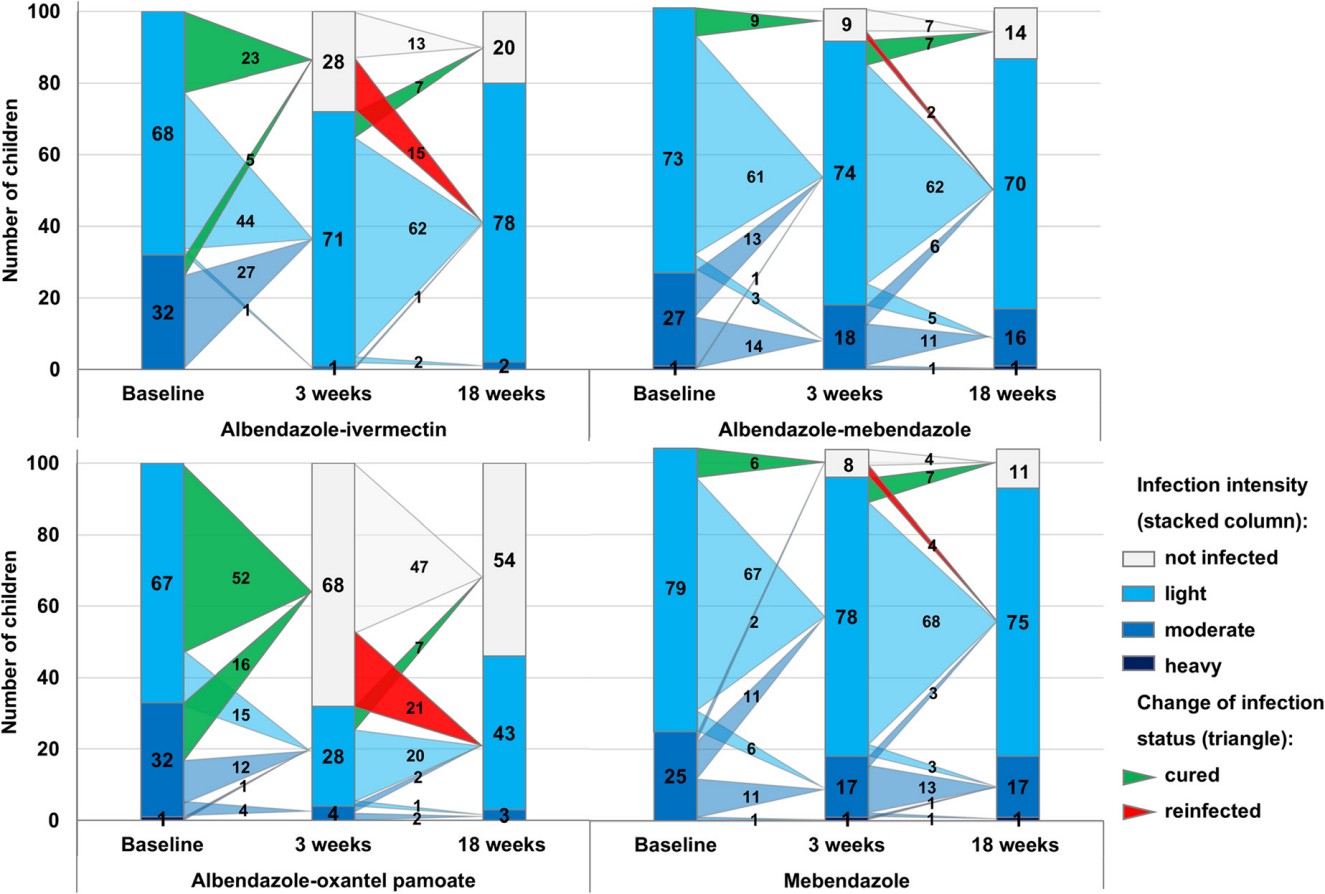
Changes in *T. trichiura* infection patterns and infection intensities from the baseline to 3 and 18 weeks post-treatment with the four different treatments

Baseline infection intensities among treatment arms were equally balanced; 70.9 % of all children harboured light, 28.6 % moderate and 0.5 % heavy infections, stratified according to The World Health Organisation (WHO) cut-offs [18]. The number of children with light, moderate and heavy infection intensities at baseline, 3 and 18 weeks post-treatment for each treatment arm are summarised in Fig. 2. Albendazole plus ivermectin and albendazole plus oxantel pamoate caused a higher reduction of moderate *T. trichiura* infections 3 and 18 weeks post-treatment compared to albendazole-mebendazole and mebendazole. At the 18 weeks follow-up, the number of moderate infections remained higher for albendazole-mebendazole (*n* = 16) and mebendazole (*n* = 17) unlike albendazole-ivermectin (*n* = 2) and albendazole-oxantel pamoate (*n* = 3; see Fig. 2).

### *T. trichiura* reinfection dynamics

For *T. trichiura* the prevalence at baseline was 100 % by design, as only *T. trichiura*-positive children were included (*n* = 405). In total 42 of 113 children (37.2 %, 28.3–46.8), were reinfected with *T. trichiura* 18 weeks after treatment. All reinfections were of mild infection intensity (Table 1, Fig. 2).

### Efficacy against *A. lumbricoides* and reinfection dynamics

At baseline, 169 (41.7 %) out of 405 children were infected with *A. lumbricoides*. All treatment arms achieved high CRs at the first follow up (Table 2) [13]. CRs decreased for all treatment arms 18 weeks post-treatment, ranging from 60.5 % for mebendazole up to 69.6 % for albendazole-ivermectin. While at the first follow-up nearly all eggs were cleared (ERR 99.8–100.0 %), 18 weeks after treatment the ERRs remained significantly lower (99.0–99.2 %), except for albendazole-ivermectin (99.7 %).

**Table 2:** Cure rates (CR), extended CRs egg-reduction rates (ERR), extended ERRs and reinfection data for the four different treatments against *A. lumbricoides* infections. Results of baseline and 3 weeks follow-up have been reported elsewhere [13]

*Ascaris lumbricoides*					
	Weeks post-treatment	Albendazole – ivermectin (*n* = 100)	Albendazole – mebendazole (*n* = 101)	Albendazole – oxantel pamoate (*n* = 100)	Mebendazole (*n* = 104)
Children positive before treatment (%)		46 (46.0)	36 (35.6)	44 (44.0)	43 (41.3)
No. of children cured (CR, 95 % CI)	3 weeks	45 (97.8, 88.5–99.9)	36 (100, 90.3–100.0)	43 (97.7, 88.0–99.9)	41 (95.4, 84.2–99.4)
No. of children negative (extended CR, 95 % CI)	18 weeks	32 (69.6, 54.2–82.3)	25 (69.4, 51.9–83.7)	27 (61.4, 45.5–75.6)	26 (60.5, 44.4–75.0)
Geometric mean: EPG	Baseline	2,385.8	1,195.3	1,503.4	1,095.2
	3 weeks	0.1	0.0	0.2	0.4
	18 weeks	6.9	9.1	12.5	10.6
ERR (95 % CI)	3 weeks	99.9 (99.9–100.0)	100 (−)	99.9 (99.9–100)	99.9 (99.8–100)
Extended ERR (95 % CI)	18 weeks	99.7 (99.1–99.9)	99.2 (97.5–99.8)^a^	99.2 (97.1–99.8)^a^	99.0 (97.7–99.6)^a^
No. of children positive (prevalence, 95 % CI)	Baseline	46 (46.0, 36.1–55.9)	36 (35.6, 26.1–45.1)	44 (44.0, 34.1–53.9)	43 (41.3, 31.7–51.0)
	3 weeks	1 (1.0, −1.0–3.0)	2 (2.0, −0.8–4.7)	3 (3.0, −0.4–6.4)	2 (2.0, −0.8–4.6)
	18 weeks	24 (24.0, 15.5–32.5)	28 (27.7, 18.8–36.6)	33 (33.0, 23.6–42.4)	33 (31.7, 22.6–40.8)
Reinfections (%, 95 % CI)	18 weeks	14/45 (31.1, 18.2–46.6)	11/36 (30.6, 16.3–48.1)	16/43 (37.2, 23.0–53.3)	16/41 (39.0, 24.2–55.5)
New infections (%, 95 % CI)	18 weeks	10/54 (18.5, 9.3–31.4)	17/63 (27.0, 16.6–39.7)	15/54 (27.8, 16.5–41.6)	16/61 (26.2, 15.8–39.1)

Data are n (%, 95 % CI) unless otherwise indicated. EPG = egg per gram of stool

^a^Significantly lower ERR compared to the 3 weeks ERR (no overlapping confidence interval assumption)

**Fig. 3:**
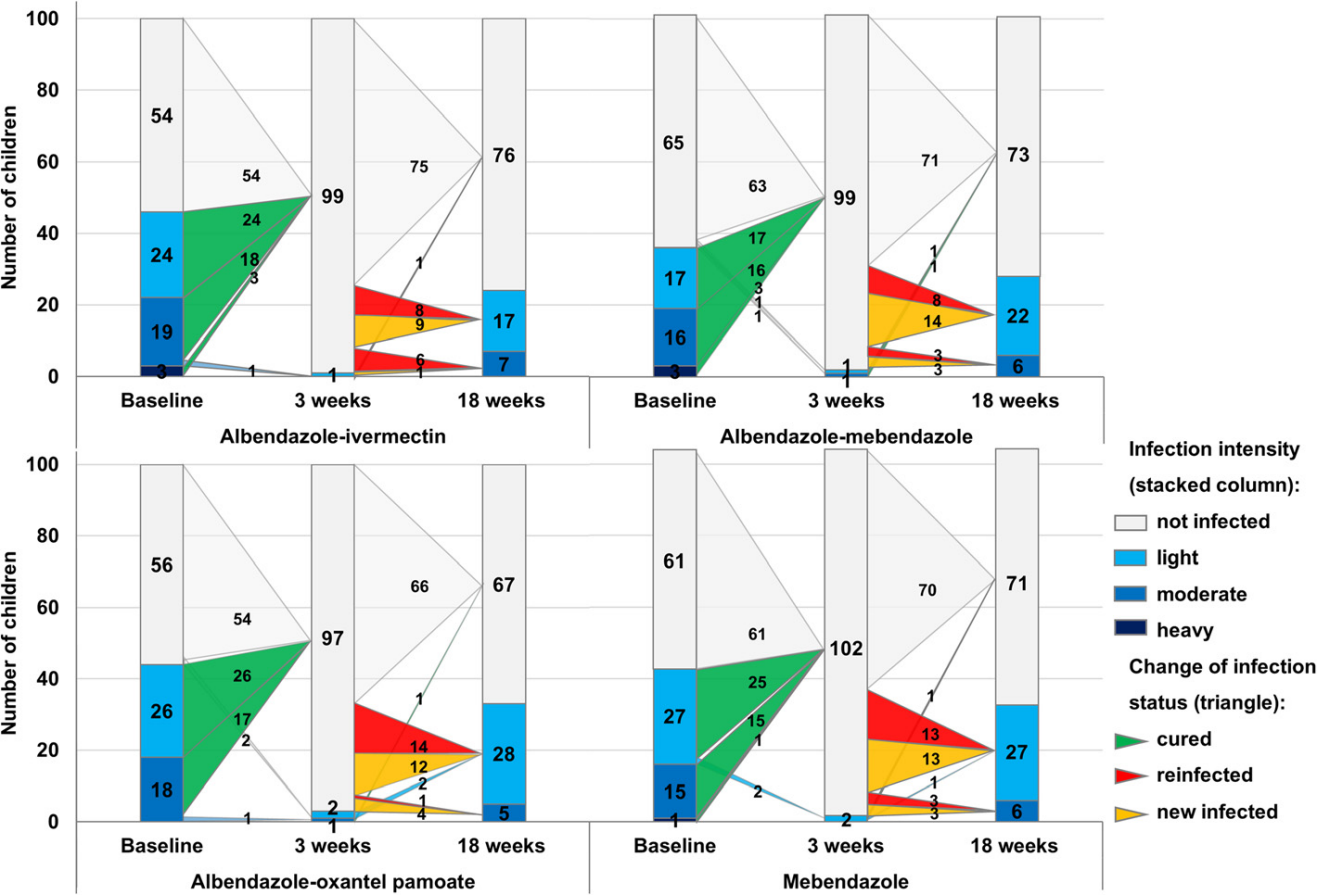
Changes in *A. lumbricoides* infection patterns and infection intensities from the baseline to 3 and 18 weeks post-treatment with the four different treatments

In total, 57 of the 165 (34.6 %; 27.3–42.3) cured children were found to be reinfected 18 weeks after being treated. Reinfections included light (*n* = 41) and moderate (*n* = 16) infections (Table 2, Fig. 3). In total 58 out of 232 (25.0 %; 19.7–31.1) children had acquired a new light (*n* = 50) or moderate (*n* = 8) *A. lumbricoides* infection.

### Efficacy against hookworms and reinfection dynamics

Hookworm prevalence among the children included in the trial was 42.5 % (233 of 405 children). At the second follow-up, slightly higher CRs were observed in all treatment groups compared to 3 weeks post-treatment: 54.0 % for albendazole-oxantel pamoate, 53.5 % for albendazole-mebendazole, 50.0 % for albendazole-ivermectin and 34.5 % for mebendazole (Table 3). At this examination time point albendazole-ivermectin (94.4 %; 88.8–97.5) and albendazole-mebendazole (95.5 %; 91.5–97.8), achieved significantly higher ERRs in comparison to mebendazole (74.1 %; 52.8–86.4). The arithmetic ERRs showed comparable results (Additional file 1: Table S1).

**Table 3:** Cure rates (CR), extended CRs, egg-reduction rates (ERR), extended ERRs and reinfection data for the four different treatments against hookworms infections. Results of baseline and 3 weeks follow-up have been reported elsewhere [13]

Hookworms					
	Weeks post-treatment	Albendazole – ivermectin (*n* = 100)	Albendazole – mebendazole (*n* = 101)	Albendazole – oxantel pamoate (*n* = 100)	Mebendazole (*n* = 104)
Children positive before treatment (%)		38 (38.0)	43 (42.6)	50 (50.0)	41 (39.4)
No. of children cured (CR, 95 % CI)	3 weeks	17 (44.7, 28.6–61.7)	21 (48.8, 33.3–64.5, *p* = 0.022)*	24 (48.0, 33.7–62.6, *p* = 0.023)*	10 (24.4, 12.4–40.3)
No. of children negative (extended CR, 95 % CI)	18 weeks	19 (50.0, 33.4–66.6)	23 (53.5, 37.7–68.8)	27 (54.0, 39.3–68.2)	14 (34.2, 20.1–50.6)
Geometric mean: EPG	Baseline	113.1	139.8	87.2	80.0
	3 weeks	6.1	8.3	7.1	31.8
	18 weeks	6.3	6.3	6.6	20.7
ERR (95 % CI)	3 weeks	94.6 (89.2–97.6)^a^	94.1 (88.7–97.0)^a^	91.9 (85.0–95.8)^a^	60.3 (27.8–79.2)
Extended ERR (95 % CI)	18 weeks	94.4 (88.8–97.5)^a^	95.5 (91.5–97.8)^a^	92.4 (85.4–96.3)	74.1 (52.8–86.4)
No. of children positive (prevalence, 95 % CI)	Baseline	38 (38.0, 28.3–47.7)	43 (42.6, 32.8–52.4)	50 (50.0, 40.0–60.0)	41 (39.4, 29.9–49.0)
	3 weeks	26 (26.0, 17.3–34.7)	26 (25.7, 17.1–34.4)	31 (31.0, 21.8–40.2)	44 (42.3, 32.7–52.0)
	18 weeks	27 (27.0, 18.1–35.9)	32 (31.7, 22.5–40.9)	34 (34.0, 24.6–43.4)	39 (37.5, 28.0–47.0)
Reinfections (%, 95 % CI)	18 weeks	5/17 (29.4, 10.3–56.0)	4/21 (19.0, .4–41.9)	6/24 (25.0, 9.8–46.7)	3/10 (30.0, 6.7–65.4)
New infections (%, 95 % CI)	18 weeks	6/57 (10.5, 4.0–21.5)	9/54 (16.7, 7.9–29.3)	8/45 (17.8, 8.0–32.1)	6/50 (12.0, 4.5–24.3)

Data are n (%, 95 % CI) unless otherwise indicated. EPG = egg per gram of stool

*Significantly higher CR compared to mebendazole (*P*-values derived from logistic regression)

^a^Significantly higher ERR compared to mebendazole (no overlapping confidence interval assumption)

**Fig. 4:**
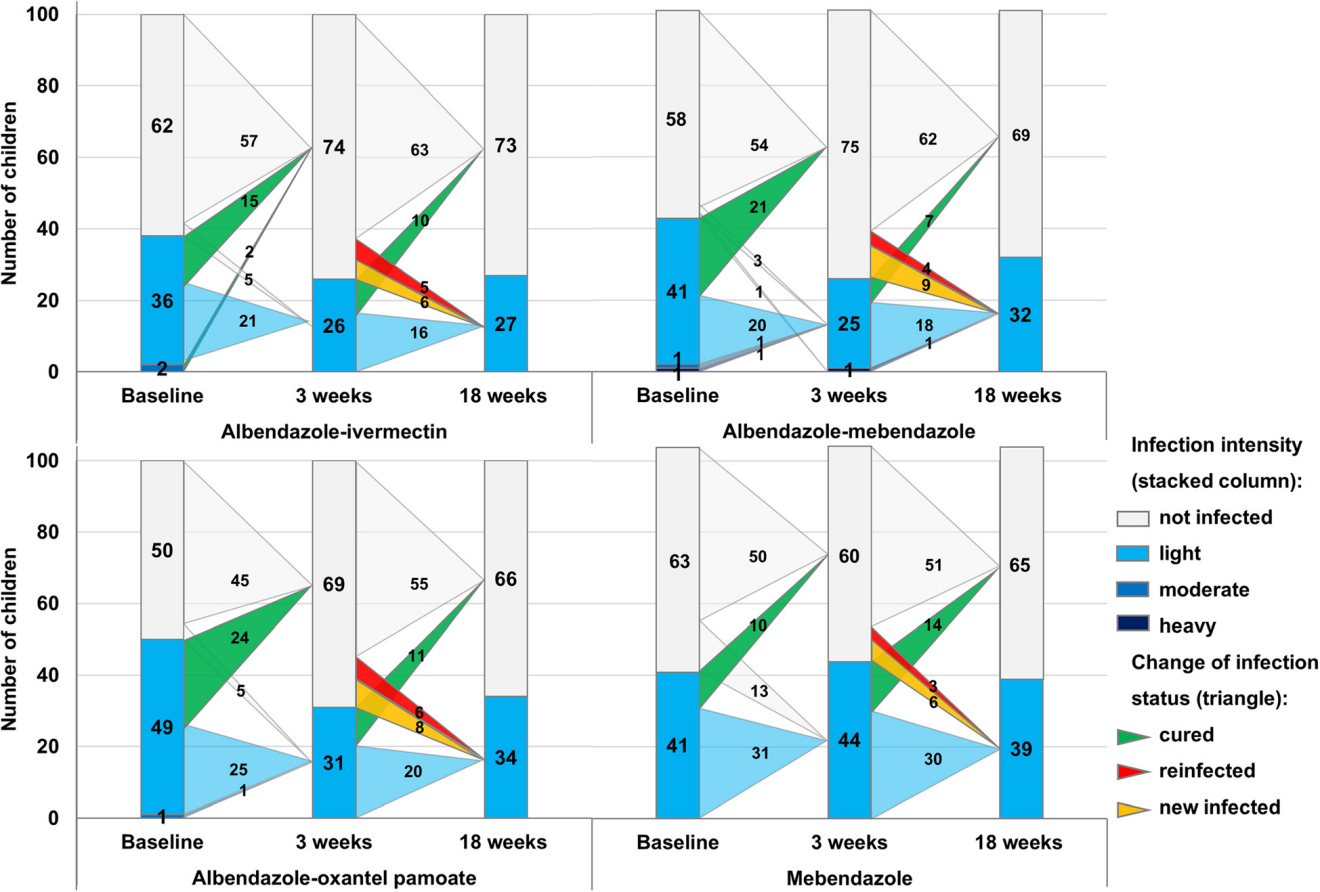
Changes in hookworms infection patterns and infection intensities from the baseline to 3 and 18 weeks post-treatment with the four different treatments

Eighteen weeks post-treatment 18 out of 72 (25.0 %; 15.5–36.6) children were reinfected with hookworms. All infections were of mild intensity (Table 3, Fig. 4). In total, 29 out of 206 children (14.1 %, CI 9.6–19.6) had acquired a new infection, ranging from 10.5 % (albendazole-ivermectin) to 17.8 % (albendazole-oxantel pamoate).

## Discussion

In preventive chemotherapy control programs albendazole and mebendazole are the treatment of choice against infections with all three STHs, yet both reveal a poor efficacy against *T. trichiura* [6]. In the recent past, oxantel pamoate has emerged as frontrunner for the treatment of infections with *T. trichiura* [13, 19, 20]. In more detail, in our recent studies albendazole-oxantel pamoate revealed good CRs and high ERRs*,* while mebendazole achieved CRs of 11.8 % [19] and 8.4 % [13] against *T. trichiura* infection. This low efficacy of mebendazole in the Pemba setting might be due to the occurrence of drug resistance, although molecular studies were not carried out to demonstrate it. In this scenario that is common also to other STH endemic areas, the need of new drug combinations in order to expand the armamentarium of treatments available for preventive chemotherapy strategy is of utmost importance [21].

In addition, albendazole-oxantel pamoate achieved a high reduction of moderate *T. trichiura* infections (persisting at 18 weeks post-treatment) in contrast to mebendazole. Note that, the goal of preventive chemotherapy is to reduce the morbidity from STH in pre- and school-aged children, by decreasing moderate and heavy infection intensities to a level below 1 % [22]. The combination albendazole-oxantel pamoate might contribute to achieve this goal.

Infections after treatment re-appear fast, particularly for *A. lumbricoides* and hence have a huge impact on the success of preventive chemotherapy [9]. This study presents detailed insights about the impact of drug combinations including the effective oxantel pamoate combination on the reinfection dynamics of the three STHs in a highly endemic area on Pemba Island, Tanzania. Earlier studies on reinfection struggled with the low CR of the standard drugs against *T. trichiura* which complicated drawing sound conclusions against this helminth species [9, 23].

Previous studies on reinfection mainly described the re-acquired level of infection after treatment, in comparison to the pre-treatment level and presented the prevalence risk ratio [9, 24, 25]. With the focus only on prevalence before and after treatment, new infections are falsely considered as reinfection. Furthermore, prevalence risk ratios are strongly influenced by the achieved CRs of the treatments. Hence, in this study we distinguish and present both; reinfections rates (positive children at baseline, negative at the 3 week examination point and positive 18 weeks after treatment) and new infections (children with an infection exclusively 18 weeks post-treatment). Note that, children negative at baseline and positive 3 weeks later, which was observed for *A. lumbricoides* and hookworms, were not considered as new infections. We assume, that these children either harboured a non-patent infection or were wrongly diagnosed as negative at baseline [26]. We are confident that our differentiation between reinfections and new infections holds true given that the majority of *A. lumbricoides* and hookworm infections were of moderate intensities at baseline. However, obviously the low sensitivity of the Kato-Katz technique mainly for light infection intensities [26–28] reflects a general limitation of our study. Please note that in the present study four slides were examined by Kato-Katz. However, even when examining multiple thick smears the Kato-Katz method only reaches moderate sensitivity [29]. Therefore our results (i.e. re-infection, new infection) have to be interpreted with caution.

Earlier studies on reinfection with *T. trichiura* reported prevalence to pre-treatment level between 6 and 12 months [9–11, 24, 30]. In our study, the overall reinfection rate 18 weeks post-treatment for *T. trichiura* was 37.2 %, which is comparable to the 3 month post treatment prevalence risk ratio of 36.0 % reported by Sinniah [31] and 39.7 % by Al-Mekhlafi et al. [24]. A similar reinfection rate was observed for *A. lumbricoides* (34.6 %), which is in agreement with the 3 month post-treatment data of Jia et al. [9]. On the other hand, Yap et al. [30] documented a higher reinfection rate 4 months after treatment. Additionally, 58 (25 %) of the children negative at baseline acquired a new *A. lumbricoides* infection 18 weeks post-treatment. Interestingly, 24 children (6.0 %) who were negative 3 weeks post-treatment harboured already a moderate infection 18 weeks post-treatment, indicating the fast reinfection potential of *A. lumbricoides* [22].

The three drug combinations cured nearly half of the hookworm-infected children 3 weeks post-treatment, while as expected mebendazole achieved only low CRs [32–35]. Surprisingly, the extended CRs and ERRs among all treatment arms (except the ERR from albendazole-ivermectin) increased at the second compared to the first follow-up. For example, in the mebendazole group, 14 children identified as hookworm-positive 3 weeks post-treatment were negative at the second follow up. This finding is most likely due to a diagnostic issue, i.e. the low sensitivity of Kato-Katz for low egg-counts [36] and a delayed reading of the microscope slides [15, 37], which could have led to fluctuations of Kato-Katz results. Overall, the reinfection rate was slower and less new infections with hookworms were observed compared to *A. lumbricoides* and *T. trichiura*, which is in agreement with other studies [9].

## Conclusions

In conclusion, our study has reconfirmed the excellent efficacy of an albendazole-oxantel pamoate combination against *T. trichiura* infections. This combination could become a key element in controlling STH infections, especially in highly endemic settings. Further trials, should evaluate reinfection rates with oxantel pamoate six and 12 month after treatment and ideally after several rounds of treatment.

The moderate reinfection rate observed for *T. trichiura* and *A. lumbricoides* is worrying. This finding supports the necessity of an integrated control approaches including regular treatment, improved sanitation and health education [38–41], in order to reduce the burden of STH infections.

## Additional file

Additional file 1: Table S1. Arithmetic mean egg per gram (EPG), egg-reduction rates (ERR) and extended ERRs for the four different treatments against *T. trichiura*, *A. lumbricoides* and hookworms infections. (DOCX 17 kb)

## Abbreviations

AM: arithmetic mean
CR: cure rate
DALYs: disability-adjusted life years
ERR: egg-reduction rate
GM: geometric mean
STH: soil-transmitted helminth
WHO: World Health Organisation
