# Supplementary material for: Efficacy and reinfection with soil-transmitted helminths 18-weeks post-treatment with albendazole-ivermectin, albendazole-mebendazole, albendazole-oxantel pamoate and mebendazole
Source: Parasit Vectors. 2016 Mar 2;9:123. doi: 10.1186/s13071-016-1406-8 (PMC4776366; doi:10.1186/s13071-016-1406-8)
Supplement: Additional file 1: Table S1. — Arithmetic mean egg per gram (EPG), egg-reduction rates (ERR) and extended ERRs for the four different treatments against T. trichiura, A. lumbricoides and hookworms infections. (DOCX 17 kb) [file 13071_2016_1406_MOESM1_ESM.docx]

**Additional file 1**

**Efficacy and reinfection with soil-transmitted helminths 18-weeks post-treatment with albendazole-ivermectin, albendazole-mebendazole, albendazole-oxantel pamoate and mebendazole**

Benjamin Speich^1,2¶^, Wendelin Moser^1,2¶^, Said M Ali^3^, Shaali M Ame^3^, Marco Albonico^4^, Jan Hattendorf^2,5^ and Jennifer Keiser^1,2*^

**1** Department of Medical Parasitology and Infection Biology, Swiss Tropical and Public Health Institute, Basel Switzerland

**2** University of Basel, Basel, Switzerland

**3** Laboratory Division, Public Health Laboratory-Ivo de Carneri, Chake Chake, Tanzania

**4** Ivo de Carneri Foundation, Milano, Italy

**5** Department of Epidemiology and Public Health, Swiss Tropical and Public Health Institute, Basel, Switzerland

*Corresponding author

E-mail: [jennifer.keiser@unibas.ch](mailto:jennifer.keiser@unibas.ch)

^¶^These authors contributed equally to this work

**S1 Table.**

| ***Trichuris trichiura*** | | | | | |
| --- | --- | --- | --- | --- | --- |
|  | Weeks post-treatment | Albendazole – ivermectin  (n=100) | Albendazole – mebendazole  (n=101) | Albendazole – oxantel pamoate (n=100) | Mebendazole (n=104) |
| Arithmetic mean – EPG | baseline | 1043.1 | 1101.0 | 1204.3 | 974.6 |
|  | 3 weeks | 127.8 | 671.0 | 151.6 | 884.2 |
|  | 18 weeks | 176.5 | 743.8 | 92.8 | 666.9 |
| ERR (95% CI) | 3 weeks | 87.7^±^ (82.6-91.6) | 39.1 (9.4-60.0) | 87.4^±^ (76.0-94.8) | 9.3 (-20.5-36.0) |
| Extended ERR (95% CI) | 18 weeks | 83.1^±^ (76.9-87.8) | 32.4 (-18.0-60.6) | 92.3^±^ (88.2-95.4) | 31.6 (-4.1-54.1) |
| ***Ascaris lumbricoides*** | | | | | |
|  | Weeks post-treatment | Albendazole – ivermectin  (n=100) | Albendazole – mebendazole  (n=101) | Albendazole – oxantel pamoate (n=100) | Mebendazole (n=104) |
| Arithmetic mean – EPG | Baseline | 13220.1 | 13635.9 | 7951.8 | 7632.0 |
|  | 3 weeks | 0.1 | 0.0 | 278.3 | 94.5 |
|  | 18 weeks | 1469.7 | 1590.0 | 1766.8 | 1312.9 |
| ERR (95% CI) | 3 weeks | 99.9 (99.9-100.0) | 100.0 (-) | 96.5 (87.4-100.0) | 98.8 (95.2-100.0) |
| Extended ERR (95% CI) | 18 weeks | 88.9^‡^ (74.2-96.8) | 88.3^‡^ (70.4-96.9) | 77.8 (43.9-96.2) | 82.8 (58.7-96.7) |
| **Hookworm** | | | | | |
|  | Weeks post-treatment | Albendazole – ivermectin  (n=100) | Albendazole – mebendazole  (n=101) | Albendazole – oxantel pamoate (n=100) | Mebendazole (n=104) |
| Arithmetic mean – EPG | Baseline | 353.1 | 390.9 | 229.6 | 173.1 |
|  | 3 weeks | 38.8 | 71.9 | 67.8 | 153.2 |
|  | 18 weeks | 58.7 | 45.9 | 101.6 | 131.0 |
| ERR (95% CI) | 3 weeks | 89.0^±^ (68.6-96.5) | 81.6^±^ (57.1-92.1) | 70.5^±^ (57.2-81.8) | 11.5 (-57.5-53.1) |
| Extended ERR (95% CI) | 18 weeks | 83.4^±^ (57.4-94.4) | 88.3^±^ (74.2-94.0) | 55.7 (40.2-77.4) | 24.3 (-1.3-45.9) |

Data are n; n (%, 95% CI); unless otherwise indicated. EPG=egg per gram of stool

^±^ Significantly higher compared to mebendazole (no overlapping confidence interval assumption)

^‡^ Significantly lower compared to the 3 weeks CRs (no overlapping confidence interval assumption)
